# Discovery of a new species of hermit crab of the genus *Pylopaguropsis* Alcock, 1905 from the Caribbean: “den commensal” or “cleaner”? (Crustacea, Anomura, Paguridae)

**DOI:** 10.3897/zookeys.646.11132

**Published:** 2017-01-19

**Authors:** Rafael Lemaitre

**Affiliations:** 1 Department of Invertebrate Zoology, National Museum of Natural History, Smithsonian Institution, 4210 Silver Hill Road, Suitland, MD 20746, USA

**Keywords:** Bonaire, Caribbean, “cleaner”, “den commensal”, hermit crab, new species, Paguridae, Pylopaguropsis

## Abstract

A new secretive, yet brightly colored hermit crab species of the family Paguridae, *Pylopaguropsis mollymullerae* **sp. n.**, is fully described based on specimens from the reefs of Bonaire, Lesser Antilles, southern Caribbean Sea. Populations of this new species were discovered and photographed in the Bonaire National Marine Park under a large coral ledge, at a depth of 13.7 m, living in crevices known by scuba divers to serve as den to a pair of “flaming reef lobsters” *Enoplometopus antillensis*, or a “broad banded moray” *Channomuraena vittata*. This new species is only the second species of *Pylopaguropsis* Alcock, 1905 known from the western Atlantic, the 20^th^ named worldwide, and belongs in the *teevana* group of species of the genus. It is remarkably similar, and herein considered geminate, to the tropical eastern Pacific congener, *Pylopaguropsis teevana* (Boone, 1932), the two being characterized and uniquely different from all other species of the genus, by the striking and deeply excavated, scoop-like ventral surface of the chela of the right cheliped. Minor differences separate this new species from *Pylopaguropsis teevana* in the relative length of the antennal acicles (exceeding the corneas versus not exceeding the corneas in *Pylopaguropsis teevana*); dorsal armature of the right chela (smooth or with scattered minute tubercles versus with numerous small tubercles in *Pylopaguropsis teevana*); surface shape of the lateral face of the dactyl of right pereopod 3 (evenly convex versus flattened in *Pylopaguropsis teevana*); and coloration (red bright red stripes versus brown stripes in *Pylopaguropsis teevana*). The highly visible color pattern of bright red stripes on white background typical of decapods known to have cleaning symbioses with fish, dense setation on the flagella of the antennae, and preference for a crevicular habitat, combined with brief in situ nocturnal observations, suggests the possibility that *Pylopaguropsis mollymullerae* sp. n. engages in “cleaner” activities or functions as a “den commensal” with moray eels. The morphology and possible meaning of the observed behavior is discussed. A tabular summary of the distribution, habitat, and published information on all species of *Pylopaguropsis* is presented. Supplemental photographs and a video of live *Pylopaguropsis mollymullerae* **sp. n.** are included.

## Introduction

The genus *Pylopaguropsis* Alcock, 1905, currently includes a group of 19 morphologically striking species that typically live in hard bottoms on or near coral reefs, and at depths ranging from the subtidal to the upper continental slope (0–610 m). One additional species remains undescribed (Arima 2014, Komai pers. comm.). Most species are distributed in the Indo-West Pacific region to Central and South Pacific as far east as Easter Island, with only two species known so far from elsewhere: *Pylopaguropsis teevana* (Boone, 1932), from the eastern tropical Pacific, and *Pylopaguropsis atlantica* Wass, 1963, from the tropical western Atlantic. In addition to their distinctive coloration patterns that typically consist of dark stripes or brightly colored appendages (Asakura 2000, Asakura and Paulay 2003, Komai and Osawa 2004, Osawa and Okuno 2007, McLaughlin et al. 2010, Asakura 2010, Rahayu and Komai 2013, Arima 2014), species of this genus stand out by having a characteristically massive right cheliped, with a large operculate or semioperculate chela, and a dactyl that articulates obliquely with the palm. Other defining diagnostic characters include 13 pairs of biserial phyllobranchiate gills; crista dentata of third maxilliped with accessory tooth; male with unpaired left pleopods 3–5; and females with paired pleopods 1 modified as gonopods, and unpaired left pleopods 2–5 (for full diagnosis see Asakura 2000: 72, and McLaughlin 2003: 126).

The taxonomic history of *Pylopaguropsis* was discussed by McLaughlin and Haig (1989) in a review of this genus which they divided into the *magnimanus* group (species with dactyls of left and right pereopods 3 dissimilar), and the *teevana* group (species with dactyls of left and right pereopods 3 similar). Briefly, this genus was originally described by Alcock (1905) as monotypic, to accommodate *Pylopaguropsis magnimanus* Henderson, 1896, a species that Alcock found to be related to, but that differed significantly from species of *Pylopagurus* A. Milne Edwards & Bouvier, 1891. A second species was added to this genus by Forest (1955), who concluded that *Eupagurus* (= *Pagurus*) *zebra* Henderson, 1896 belonged in *Pylopaguropsis*. Subsequently, a third species and first from the Atlantic, was added, *Pylopaguropsis atlantica* Wass, 1963. McLaughlin and Haig (1989) found that the monotypic *Galapagurus* Boone, 1932 agreed with the more senior *Pylopaguropsis*, proceeded to synonymize the two genera, and thus changed the genus of its single eastern Pacific species, *Galapagurus teevanus* Boone, 1932, as well as the spelling of Boone’s species name to *teevana* in order to agree with the feminine *Pylopaguropsis*. McLaughlin and Haig also added seven new species, all from the Indo-West to the Central or South Pacific islands, bringing to 11 the total number of species of *Pylopaguropsis* known at the time. In a review of Japanese species of *Pylopaguropsis*, Asakura (2000) described two more new species from Japan, and later Asakura and Paulay (2003) added yet another new species from French Polynesia. Komai and Osawa (2004), and Osawa and Okuno (2007) each added one more new species from Japanese waters. Finally, three more new species were described from the Philippines, one by Asakura (2010), two by Rahayu and Komai (2013), and as previously mentioned, yet another one remains undescribed (Arima 2014). Although all known species of *Pylopaguropsis* are now well documented from a taxonomic and morphological point of view, their biology and ecology is at best poorly known.

Recent underwater photographs and video obtained using scuba by Ms Ellen Muller at several dive sites in the National Marine Park of the southern Caribbean island of Bonaire, Lesser Antilles, revealed the presence of a small (a few millimeters in size), intriguing and brightly colored red-striped pagurid hermit crab that appeared to belong to *Pylopaguropsis*. The specimens in the photographs, however, could not be matched using images alone, to any of the known species of the genus from the western tropical Atlantic, and seemed to represent and undescribed species. A specimen of this hermit crab was first photographed inadvertently alongside an individual of the “flaming reef lobster”, *Enoplometopus antillensis* (Lütken, 1865), sighted while observing reef invertebrates that aggregate in crevices under a large coral ledge, and subsequently additional specimens of this hermit crab were again photographed in a nearby crevice inhabited by a “broad banded moray” *Channomuraena vittata* (Richardson, 1845). In order to study in detail and determine the identity of this unusual hermit crab, permits were obtained from the Government of the Island Territory of Bonaire to collect a few specimens and ship them for study to the National Museum of Natural History, Smithsonian Institution, Washington DC, USA (USNM). Close examination of the specimens collected confirmed not only that indeed they are of a new species of *Pylopaguropsis*, but also that this new species is remarkably similar and undoubtedly more closely related, to the single eastern tropical Pacific member of this genus, *Pylopaguropsis teevana*. Furthermore, the morphological characteristics, color pattern, and observed reclusive behavior in proximity to the moray eel, suggest the possibility that this new species might function in some capacity as a “cleaner”, or perhaps is ecologically associated as a commensal with the moray eel. This new species is herein fully described and illustrated, including color photographs, and video of live specimens in the habitat where it was found. A list of all known species of *Pylopaguropsis* from the world is included, with a summary of their geographic distributions, depth ranges, and recorded habitats (Table 1).

**Table 1.** List of species of *Pylopaguropsis* Alcock, 1905 from the world, with their general geographic and bathymetric distribution. CNP: Central North Pacific; EP: eastern Pacific; IO: Indian Ocean; IWP: Indo-West Pacific; RS: Red Sea; SEP: southeastern Pacific; WA: western Atlantic; WP: western Pacific.

Genus/species	Geographic distribution	Depth range (m)	Habitat	References
*Pylopaguropsis atlantica* Wass, 1963	WA: southeast Florida, USA; Straits of Florida; Colombia; Suriname	84–200	coral, rock	Wass 1963, Bullis and Thompson 1965, McLaughlin and Haig 1989, Campos et al. 2005
*Pylopaguropsis bellula* Osawa & Okuno, 2007	WP: Ryukyus Islands, Japan	18–30	submarine caves or dark crevices on fore reef slopes	Osawa and Okuno 2007, Arima 2014
*Pylopaguropsis fimbriata* McLaughlin & Haig, 1989	IWP: Okinawa, Japan; Guam; east Malaysia; Indonesia	10–15	hard bottom, coral, dark crevices on rocky walls	McLaughlin and Haig 1989, Asakura 2000, Arima 2014
*Pylopaguropsis furusei* Asakura, 2000	WP: Ogasawara Islands, Japan	3–30	hard bottom, coral, crevices on rocky walls	Asakura 2000, Arima 2014
*Pylopaguropsis garciai* McLaughlin & Haig, 1989	SEP: Easter Island, Chile	40	probably coral or hard bottom	McLaughlin and Haig 1989
*Pylopaguropsis granulata* Asakura, 2000	WP: Okinawa, Japan	10	coral, hard bottom, crevices	Asakura 2000, Arima 2014
*Pylopaguropsis keijii* McLaughlin & Haig, 1989	CNP, IWP: Hawaii; Okinawa, Japan; Guam and West Caroline Islands; Maldives; Zanzibar	10–17	coral (*Pocillopora meandrina*), crevices on rocky walls	McLaughlin and Haig 1989, Asakura 2000, Arima 2014
*Pylopaguropsis laevispinosa* McLaughlin & Haig, 1989	WP: Okinawa, Ryukyus Islands, Japan	3–70.1	probably coral or hard bottom	McLaughlin and Haig 1989, Komai and Osawa 2004
*Pylopaguropsis lemaitrei* Asakura & Paulay, 2003	SP: Marquesas and Tuamotu Archipelago, French Polynesia	4.6–12.2	under rocks on outer reef slope	Asakura and Paulay 2003
*Pylopaguropsis lewinsohni* McLaughlin & Haig, 1989	RS: Gulf Aqaba	0–10	coral, dark crevices on rocky walls	McLaughlin and Haig 1989, Arima 2014
*Pylopaguropsis magnimanus* (Henderson, 1896)	IO: Bay of Bengal to Sri Lanka; northern Arabian Sea	119–397	soft bottom (mud)	Alcock 1902, McLaughlin and Haig 1989
*Pylopaguropsis mollymullerae* sp. n.	WA: Bonaire, southern Caribbean	11.6–13.7	in crevices under coral ledges	This report
*Pylopaguropsis pustulosa* McLaughlin & Haig, 1989	IO: Somalia	90	unknown	McLaughlin and Haig 1989
*Pylopaguropsis pygmaeus* Rahayu & Komai, 2013	WP: Philippines	80–128	sand on echinoderms bed	Rahayu and Komai 2013
*Pylopaguropsis rahayuae* Asakura, 2010	WP: Philippines	4–30	reef wall with cave, reef platform and slope	Asakura 2010
*Pylopaguropsis similis* Rahayu & Komai, 2013	WP: Philippines	100	unknown	Rahayu and Komai 2013
*Pylopaguropsis speciosa* McLaughlin & Haig, 1989	WP: Okinawa, Japan	10–610	coral or hard bottom, crevices	McLaughlin and Haig 1989, Asakura 2000, Arima 2014
*Pylopaguropsis teevana* (Boone, 1932)	EP: Colombia; Ecuador; Galapagos Islands	0.3–9.7	probably coral or hard bottom	Boone 1932, McLaughlin and Haig 1989
*Pylopaguropsis vicina* Komai & Osawa, 2004	IWP: Kii Peninsula and Nansei Islands, Japan; Banda Sea, Indonesia	50–167	sponge and coral bottom, seamount	McLaughlin and Haig 1989, Komai and Osawa 2004
*Pylopaguropsis zebra* (Henderson, 1893)	IWP: Japan; Korea Strait; East China Sea; Indonesia; Australia; South Africa	50–180	coral or hard bottom, dark crevices on rocky walls	McLaughlin and Haig 1989, Asakura 2000, Arima 2014
*Pylopaguropsis* sp. (undescribed)	IWP: Japan	72	coral, rocky substrate	Arima 2014

## Materials and methods

The holotype and paratypes of the new species described herein are deposited in the collections of the National Museum of Natural History, Smithsonian Institution, Washington DC (USNM). The specimens were collected by hand, using scuba, and during late afternoon or night dives, by E Muller. General terminology follows that used by McLaughlin and Haig (1989), Lemaitre (1995), and McLaughlin (2003). Pereopods and pleopods are indicated with a number, except for the chelipeds, which correspond to pereopods 1. Measurement indicated for the specimens are of shield length, measured in millimeters (mm), taken from the midpoint of rostral lobe to midpoint of posterior margin of shield.

## Results

### Systematic account  Family Paguridae Latreille, 1802

#### Pylopaguropsis mollymullerae sp. n.

Taxon classificationAnimaliaDecapodaPaguridae

http://zoobank.org/329C796C-EC46-4A85-A9EB-3FD21298A4BC

**Figure 1.**
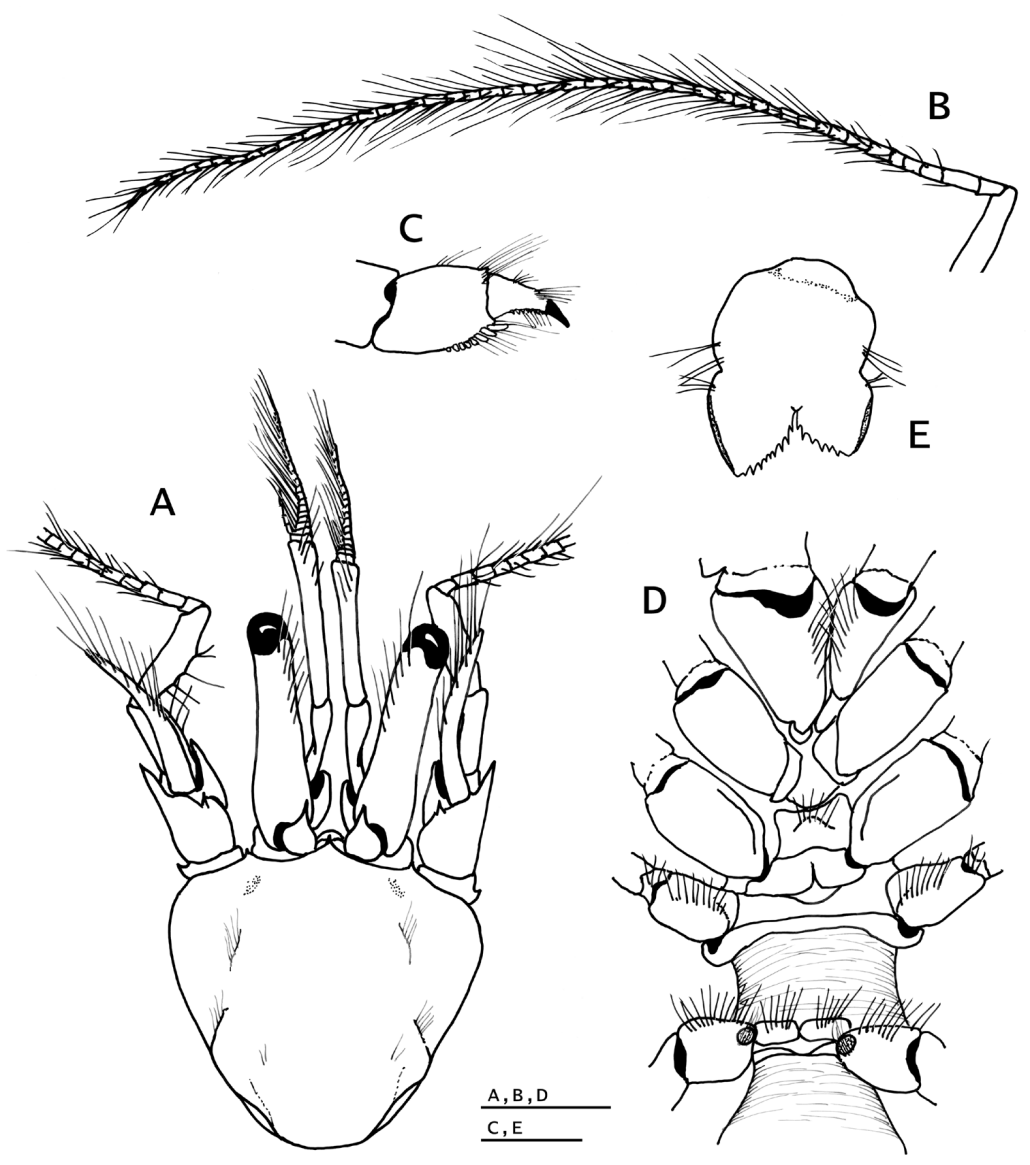
*Pylopaguropsis mollymullerae* sp. n., holotype male 2.4 mm, Bonaire (USNM 1291987). **A** shield, cephalic appendages, dorsal **B** left antennal flagellum, dorsal **C** propodus and dactyl of righ pereopod 4, lateral **D** sternum and coxae of chelipeds and pereopods 2–5, ventral **E** telson, dorsal. Scale bars 1 mm for **A, B, D**; 0.5 mm for **C, E**.

**Figure 2.**
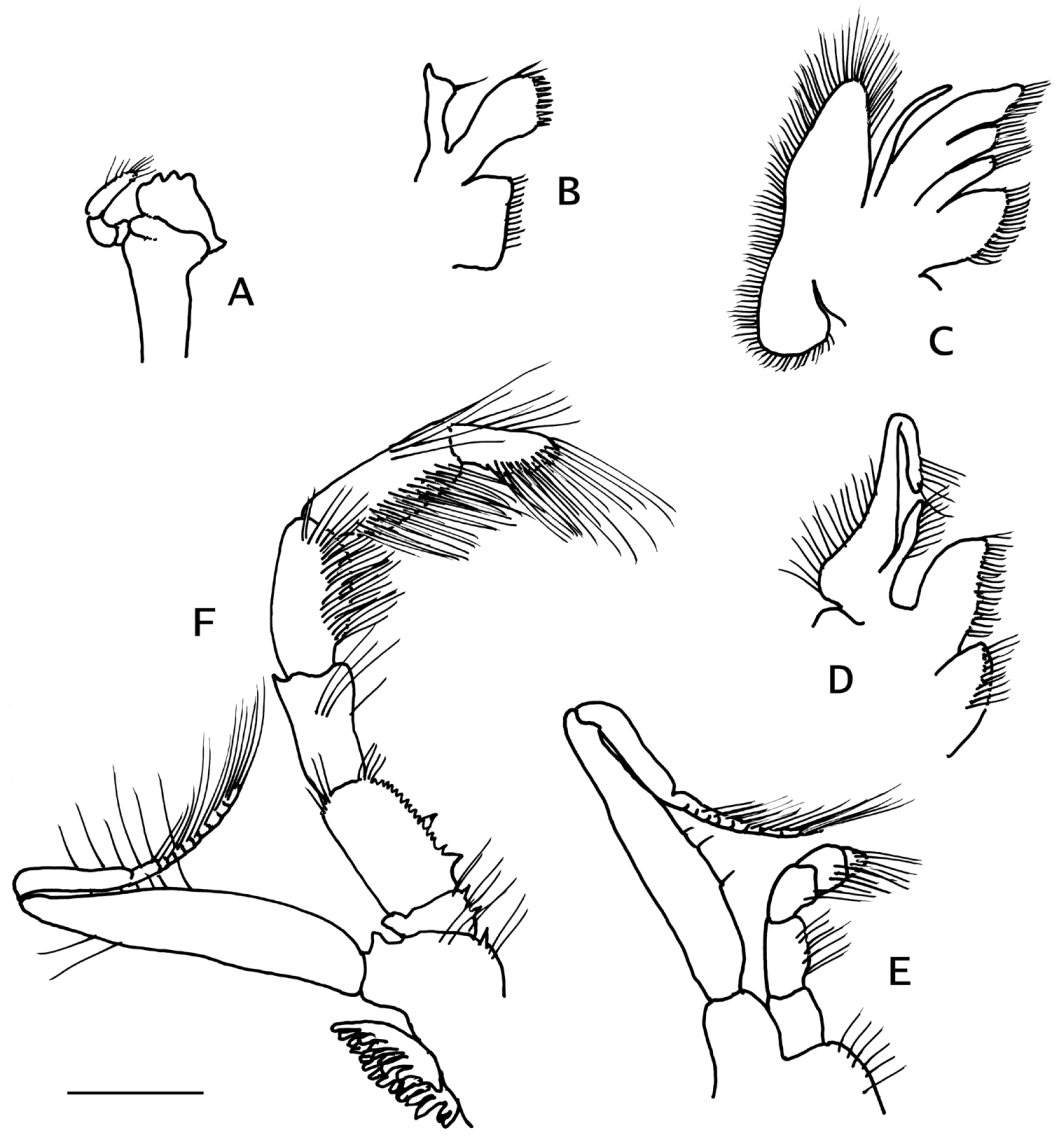
*Pylopaguropsis mollymullerae* sp. n., paratype male 1.8 mm, Bonaire (USNM 1291989). Left mouthparts, internal: **A** mandible **B** maxillule **C** maxilla **D** first maxilliped **E** second maxilliped **F** third maxilliped. Scale bar 0.5 mm.

**Figure 3.**
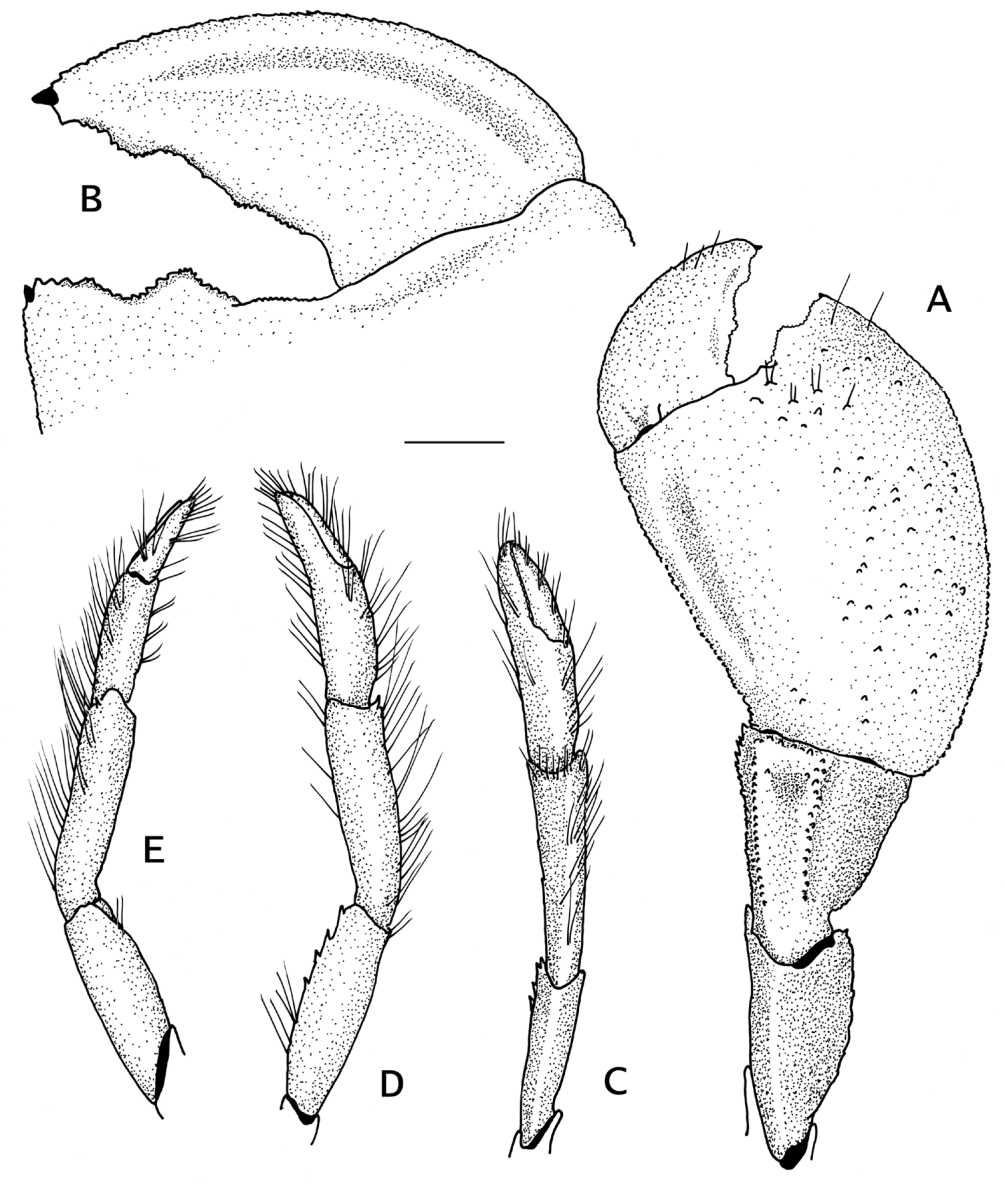
*Pylopaguropsis mollymullerae* sp. n., holotype male 2.4 mm, Bonaire (USNM 1291987). **A** right cheliped, dorsal **B** dactyl and fixed finger of same, ventral **C** left cheliped, dorsal **D** same, lateral **E** same mesial. Scale bar 1 mm.

**Figure 4.**
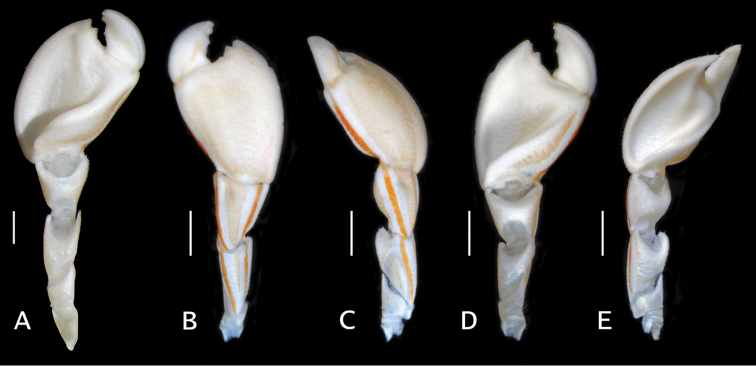
*Pylopaguropsis mollymullerae* sp. n., right cheliped. **A** ventral, holotype male 2.4 mm, Bonaire (USNM 1291987) **B–E** dorsal (**B**), dorsomesial (**C**), ventral (**D**), ventromesial (**E**), paratype male 1.8 mm, Bonaire (USNM 1291989). Scale bars 1 mm.

**Figure 5.**
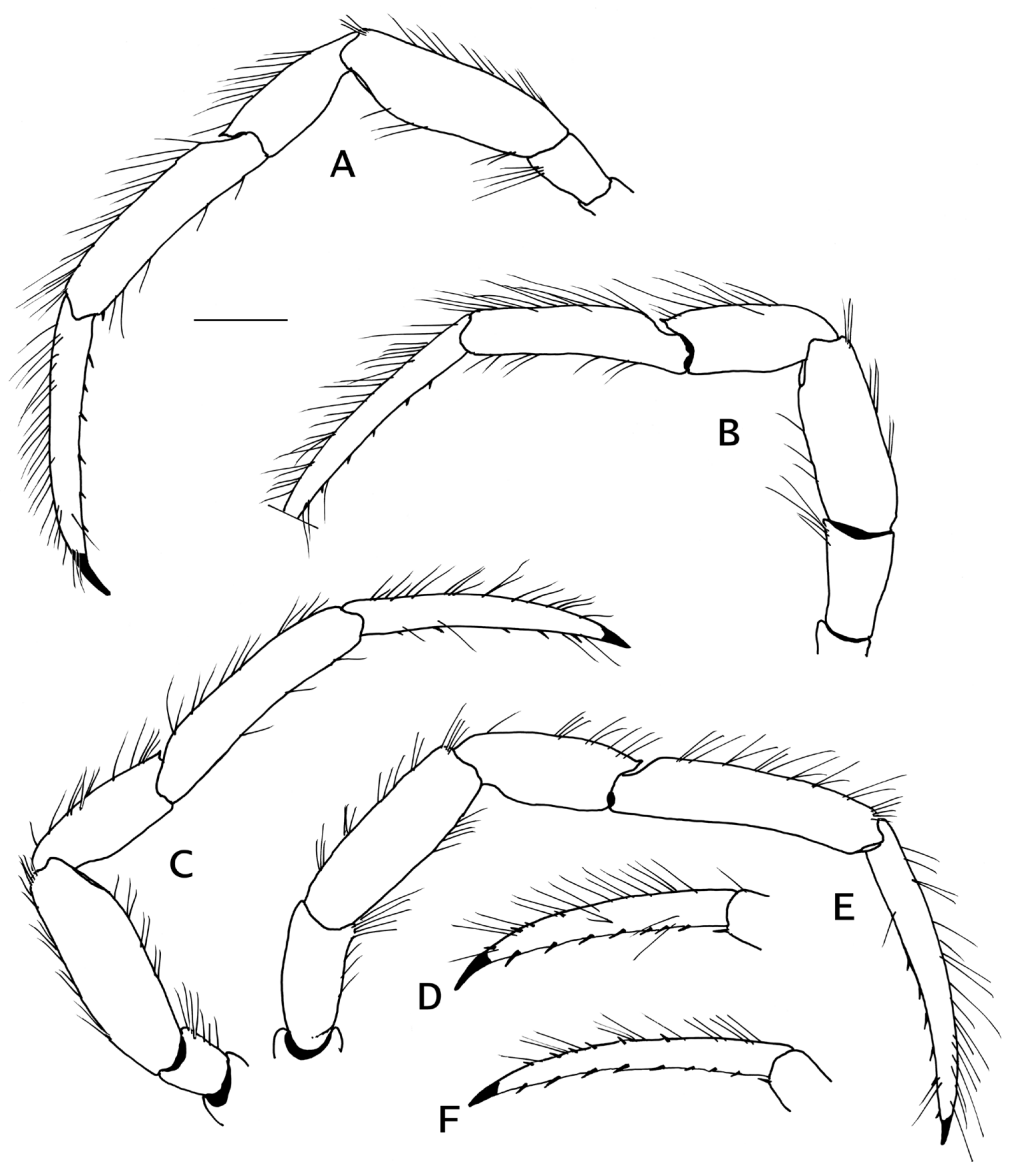
*Pylopaguropsis mollymullerae* sp. n., holotype male 2.4 mm, Bonaire (USNM 1291987). **A** left pereopod 2, lateral **B** left pereopod 3 (dactyl tip missing), lateral **C** right pereopod 2, lateral **D** dactyl of same, mesial **E** right pereopod 3, lateral **F** dactyl of same, mesial. Scale bar 1 mm.

**Figure 6.**
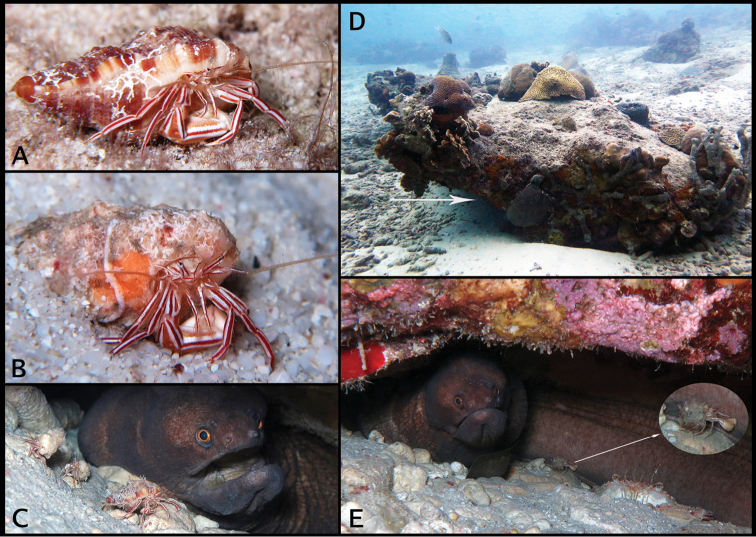
In situ photographs of *Pylopaguropsis mollymullerae* sp. n. and its habitat at Bonaire diving site “Something Special”. **A** holotype male 2.4 mm, Bonaire (USNM 1291987) **B** paratype male 1.8 mm, Bonaire (USNM 1291989) **C** three individuals of *Pylopaguropsis mollymullerae* sp. n. (foreground, not collected) in den with “broad banded moray” *Channomuraena vittata* **D** coral ledge habitat, with arrow indicating entrance to crevice where five specimens of *Pylopaguropsis mollymullerae* sp. n. were collected **E** individual of *Pylopaguropsis mollymullerae* sp. n. (expanded and enhanced in oval inset, not collected) on body surface of “broad banded moray” *Channomuraena vittata*, with frontal portion of brachyuran *Achelous sebae* visible on lower right.

Figs 1 , 2 , 3 , 4 , 5 , 6 , Table 1 , Suppl. materials 1 , 2 , 3 (color photographs, video)

##### Type material.

Holotype: male 2.4 mm, “Something Special”, central W coast of Bonaire, 12°09'46.0"N, 68°17'08.6"W, 11.6 m, 17 December 2015, sand/rubble under coral ledge, night dive, coll. E Muller (USNM 1291987).

Paratypes (all same locality, habitat, and collector, as holotype): 1 male 1.8 mm (USNM 1291989), 1 male 2.2 mm (USNM 1291988), 13.7 m; 1 male 0.9 mm, 1 female 1.5 mm, 27 January 2016 (USNM 1292072); 1 ovigerous female 1.9 mm, 28 January 2016 (USNM 1292073).

##### Non-type specimens (not collected) photographed in situ.

5 sex undertermined (Fig. 6C, E, and Suppl. material 3), "Something Special", 12°09'46.0"N, 68°17'08.6"W, ~10 m, night dive; 1 ovigerous female, 1 sex undetermined (color photos in Suppl. materials 1–2), “Front Porch”, E Muller night dives 4125 095a and 4125 156z2, central W coast of Bonaire, 12°09'55.2"N, 68°17'12.0"W, night dive, 12.2 m, 17 September 2016.

##### Description.

Shield (Fig. 1A) about as long as broad, weakly convex, well calcified, smooth except for weak depression behind each lateral projection; naked except for few short rows or tufts of fine setae. Rostrum acutely triangular, ending in small, sharp spine, and extending well beyond distal level of lateral projections. Anterior margins between rostrum and lateral projections concave. Lateral projections broadly triangular, ending in small, sharp spine. Anterolateral margins sloping. Accessory portions narrow, well calcified, fused to shield. Branchiostegite membranous; anterior margin rounded, setose.

Ocular peduncles moderately long, about 0.8 as long as shield, slightly inflated basally and tapering to base of corneas, with dorsodistal row of long setae; corneas weakly dilated. Ocular acicles subtriangular, with strong terminal spine; separated basally by less than basal width of one acicle.

Antennules (Fig. 1A) exceeding distal margins of corneas by 0.5–0.6 length of ultimate segment. Ultimate segment with few long dorsodistal setae. Penultimate and basal segments naked or with scattered short setae. Basal segment with laterodistal spine. Ventral flagellum with five or six articles.

Antennal peduncles (Fig. 1A) exceeding distal margins of corneas by 0.2 length of fifth segment. Fifth and fourth segments unarmed except for sparse setae. Third segment with strong ventromesial spine. Second segment naked or with scattered short setae; dorsolateral distal angle strongly produced, ending in small spine at tip; dorsomesial distal angle with small spine. First segment with small spine on lateral face distally. Acicle broadly curved outward, slightly exceeding distal margin of cornea, terminating in strong spine; with dorsomesial row of long setae. Flagellum (Fig. 1B) slightly exceeding extended right cheliped, densely setose, with setae >1 - 8 flagellar articles in length.

Mandible (Fig. 2A) with edge of incisor process armed with three blunt calcareous teeth. Maxillule (Fig. 2B) with endopod slender, internal lobe with one long bristle. Maxilla (Fig. 2C) with endopodite slightly exceeding distal end of scaphognathite. First maxilliped (Fig. 2D) with endopodite not exceeding distal endite. Second maxilliped (Fig. 2E) without distinguishing characters. Third maxilliped (Fig. 2F) with merus armed with small blunt, dorsodistal spine; ischium with crista dentata consisting of about 13 small, subequal corneous teeth, two larger basal teeth, and one accessory tooth; basis with two sharp teeth on mesial margin; coxa with small distomesial spine. Sternite IX (of third maxillipeds) with small, sharp spine on each side of midline.

Chelipeds markedly asymmetrical. Right cheliped (Fig. 3A, B, 4A–E) massive, nearly naked, with chela operculate, ovate; fingers curving ventrally, broad basally and terminating in small, inwardly curved, corneous claw; cutting edges (Fig. Fig. 3B) uneven, minutely and sharply denticulate, fixed finger with large subtriangular tooth medially. Dactyl 0.8 times as long as palm, set very obliquely relative to longitudinal axis of chela; dorsoventrally flattened; dorsal surface weakly convex, unarmed except for scattered short setae; dorsomesial margin sharp, plate-like; ventral surface moderately concave, with longitudinal ridge parallel to mesial margin. Palm and fixed finger weakly convex, nearly naked or with scattered short setae; dorsal surfaces with scattered small, low tubercles on lateral half, smooth except for shallow longitudinal groove on mesial half; lateral margin sharply defined by minute denticles; mesial face strongly produced ventrally, ventromesial margin rounded; ventral surface smooth, deeply excavated, mesial face and lateral margin forming distinct, semi-cylindrical scoop-like surface. Carpus subtrapezoidal, naked; dorsal surface with dorsolateral and dorsomesial ridges marked by minute, sharp spines; lateral face sloping; ventrolateral margin unarmed; mesial face strongly sloping, distomesial margin with row of minute spines; ventral surface convex, smooth. Merus subtriangular, smooth; ventromesial margin minutely spinulose proximally except for row of moderately strong spines distally; ventrolateral margin unarmed. Ischium unarmed except for ventromesial row of setae.

Left cheliped (Fig. 3C–E) slender, reaching nearly to base of dactyl of right cheliped. Fingers each terminating in short, inwardly curved corneous claw; dactyl shorter than palm, cutting edge with row of closely set, minute corneous spinules, mesial face with few tufts of long setae; fixed finger with tufts of setae on lateral face, cutting edge with row of small, well-spaced calcareous teeth interspersed with fused corneous spinules. Palm smooth, naked except for tufts of long setae mostly on dorsomesial face. Carpus subtriangular; dorsal margin with row of tufts of setae, lacking spines. Merus nearly naked except for scattered tufts of setae; ventrolateral margin with row of few well-spaced spines usually on distal half; ventromesial margin unarmed. Ischium unarmed except for ventromesial row of setae.

Ambulatory legs or pereopods 2 and 3 (Fig. 5A–F) not significantly different left from right; meri to dactyls with lateral faces evenly convex, with long setae or tufts of setae on dorsal margins. Dactyl nearly straight, about 1.8 times as long as propodi, terminating in sharp corneous claw; dorsomesial margin with row of minute corneous spinules on distal half; ventromesial margin with row of 6–8 corneous spinules. Propodus slightly arched. Carpi each with small dorsodistal spine. Merus unarmed. Ischium with row of setae on ventral margins.

Sternite XII (of pereopod 3; Fig. 1D) with anterior lobe weakly rounded, setose.

Pereopod 4 (Fig. 1C) semichelate. Dactyl subtriangular, terminating in sharp, corneous claw, lacking preungual process; with ventrolateral row of minute, fused corneous teeth. Propodal rasp consisting of one distal row of lanceolate scales.

Pereopod 5 chelate. Propodal rasp occupying nearly half of lateral face of propodus.

Sternite XIV (pereopod 5) subdivided anteriorly into two subrectangular, setose lobes (Fig. 1D).

Uropods strongly asymmetrical. Telson (Fig. 1E) with distinct lateral indentations separating anterior and posterior lobes; posterior lobes subtriangular, nearly symmetrical, separated by deep, narrow median cleft, distal margins armed with row of small, sharp spines and blunt laterodistal angle.

Male with paired gonopores (Fig. 1D), and unpaired left pleopods 2–5. Female with paired pleopods 1 modified as gonopods, and unpaired left pleopods 2–5 (pleopod 5 not ovigerous).

##### Coloration

**(Fig. 6A, B, Suppl. materials 1–3).** General background color white with bright red stripes. Shield white with two pairs of oblique red stripes on each side of anterior half. Ocular peduncles white with two red stripes uniting across corneas. Corneas transparent, with black colored core. Antennular peduncle white with red dorsal stripe. Antennal peduncles white with red dorsal and ventral red stripe; antennal acicle white with red dorsal stripe; flagellum light red. Right cheliped with dorsal surfaces of chela, carpus and merus light red, and mesial faces with continuous red stripe; lateral faces of carpus and merus white with continuous red stripe. Left cheliped white with dorsal, lateral, and mesial red stripes. Ambulatory legs or pereopods 2 and 3, with dactyl white with red lateral and mesial stripes; merus to propodus with dorsal, lateral and mesial red stripes; ischium white with red dorsal stripe.

##### Distribution.

So far known only from the island of Bonaire, Lesser Antilles, southern Caribbean Sea; depth: 11.6–13.7 m.

##### Etymology.

The name of this new species is given to acknowledge the efforts of the collector, photographer and environmentalist, Ms Ellen Muller, who when informed of the intended honor, preferred that the name of her granddaughter, Ms Molly Muller, be used, in hopes to inspire her to continue the tradition of protecting the amazing and fragile diversity of marine life in Bonaire.

##### Common name.

“Candy striped hermit crab”, in reference to the bright white and red striped color pattern that is similar to that of traditional candy cane.

##### Affinities.

*Pylopaguropsis mollymullerae* sp. n. is remarkably similar in morphology to *Pylopaguropsis teevana*, a species distributed in the tropical eastern Pacific from Colombia to Ecuador, including the Galapagos Islands. There is such minimal differentiation between the two species that they can be considered geminate. They are unique among congeners and even among other Paguridae, in the unusual structure of the right chela, with a ventral surface deeply excavated, forming a semi-cylindrical scoop-like surface (Fig. 4A, D, E). Both species also have antennal flagella with long, dense setae. The condition of the antennal flagella was not mentioned by McLaughlin and Haig (1989) in their redescription of *Pylopaguropsis teevana*, although in her description Boone (1932: fig. 4, as *Galapagurus teevanus*) did illustrate this setal condition, noting that the flagella have “long radiating setae”. The only differences detected between these two species are minor, as follows: the antennal acicles slightly exceed the corneas, whereas the acicles do not reach the distal margin of the cornea in *Pylopaguropsis teevana*; the dorsal surfaces of the right chela is smooth (dactyl) or with only scattered minute tubercles on the lateral half (palm), whereas the same surfaces have numerous albeit well-spaced small tubercles in *Pylopaguropsis teevana*; and the lateral face of the dactyl of right pereopod 3 is evenly convex, whereas the lateral surface is slightly flattened in *Pylopaguropsis teevana*. Although coloration was only briefly described by McLaughlin and Haig (1989: 159, based on AJ Provenzano, Jr’s color notes) for *Pylopaguropsis teevana*, it appears that at least the striped pattern of that eastern Pacific species is similar to that of the Caribbean *Pylopaguropsis mollymullerae* sp. n. In this new species, however, the stripes are bright red over white, whereas in *Pylopaguropsis teevana* the stripes are brown over light cream.

Given that the dactyls of the left and right pereopods 3 in *Pylopaguropsis mollymullerae* sp. n. are similar, this new species belongs in the *teevana* group of species of this genus as defined by McLaughlin and Haig (1989).

##### Habitat and behavior.

As previously mentioned, *Pylopaguropsis mollymullerae* sp. n. was first photographed fortuitously, but not collected, while observing the “flaming reef lobster” *Enoplometopus antillensis*. Five of the six specimens collected of *Pylopaguropsis mollymullerae* sp. n. were found living in gastropod shells, and obtained during dives in late afternoon or at night inside a crevice under a large coral ledge about 3.6 m wide by 1.5 m high at the site “Something Special” (Fig. 6D). The sixth specimen was collected a short distance north of that ledge, at a slightly shallower depth. Specimens were photographed, but not collected, in a crevice where a large “broad banded moray” *Channomuraena vittata*, has been known to divers to live for many years (Fig. 6C, E). An “ocellate swimming crab”, *Achelous sebae* (H. Milne Edwards, 1834), was also observed at this crevice (Fig. 6E). It appears that this new species is reclusive, and prefers to hide in the deep, dark recesses of crevices under coral ledges where divers (E Muller, pers. comm.) occasionally have also observed two other species of moray eels, the “spotted moray” *Gymnothorax moringa* (Cuvier, 1829), and the “green moray” *Gymnothorax funebris* Ranzani, 1839. After the discovery of specimens of *Pylopaguropsis mollymullerae* sp. n., additional photographs were taken at the site “Front Porch”, and a video at the site “Something Special”, in order to document any particular behavior (see Suppl. materials 1–3). Frequently, one or more individuals of this this new hermit crab species were seen in close proximity of a moray eel, and in one instance clearly on its body (Fig. 6E).

## Discussion

Several aspects of *Pylopaguropsis mollymullerae* sp. n. merit commentary. As previously noted, the morphological similarity of this new species with the eastern Pacific congener, *Pylopaguropsis teevana*, is so remarkable that there is little doubt the two are closely related and geminate species that have barely diverged since the complete ocean separation by the central American isthmus. Among the Paguridae that occur in the tropical western Atlantic–tropical eastern Pacific region, very few genera (e.g., *Phimochirus* McLaughlin, 1981, *Spathapagurus* Lemaitre & Felder, 2011) have species that have been declared to contain geminates, although the two regions share a considerable number of genera and have a close geologic history. In addition to the similarity of the deeply excavated, scoop-like ventral surface of the right chela, both species also have the antennal flagella with long setae which in life are set at about 90° angle to the axis of the flagellum (Fig. 6A, B). The function, if any, of the unusual shape of the right chela, and the antennal flagella, is intriguing. A video of an individual of *Pylopaguropsis mollymullerae* sp. n. taken in situ (see Suppl. materials 1–3) shows the hermit crab walking while maintaining the right cheliped partially retracted or bent against the body, so that the chela is positioned in a shield-like manner. When the hermit crab is in motion, the dorsal surface of the chela is facing the substrate, and is clearly used to push itself along the bottom. Despite the scoop-like shape of the ventral surface of the chela which suggests it might be used for gathering materials or maybe digging, no such uses were observed. The long antennal flagella are held straight out on the sides in wing-like fashion, and parallel to the substrate. While these observations of live crabs do not show conclusively any particular function, it seems clear that the odd morphology must represent a specialized adaptation that deserves further study.

Given that *Pylopaguropsis mollymullerae* sp. n. lives in relatively shallow (scuba depth) habitats in reefs with crystal clear waters, has a conspicuous, bright color pattern, and individuals are of sufficiently large size to be visible to the naked eye, it is surprising that this new species had not been previously discovered. If what we know of the distribution of other pagurids in the western Atlantic is any indication (species are generally broadly distributed), it is unlikely that the occurrence *Pylopaguropsis mollymullerae* sp. n. is geographically restricted to Bonaire. It seems more probable that its presence had not been detected before, in part, because of its secretive, crevicular, nocturnal behavior, apparent association with menacing moray eels that detract collectors, and preference to live in environments such as large coral ledges that are difficult to reach unless using scuba. Based on the few observations that have been made (E Muller, pers. comm.), this new species ventures out of the coral crevices only for short distances during night time. Regrettably, knowledge of the biology or ecology of other species of *Pylopaguropsis* is fragmentary at best, and thus it is not possible to make significant extrapolations or comparisons except that most species of this genus have, as previously mentioned, bright and often spectacular colorations (Asakura 2000, 2010, Asakura and Paulay 2003, Komai and Osawa 2004, Osawa and Okuno 2007, McLaughlin et al. 2010, Rahayu and Komai 2013, Arima 2014). Based on the information available (Table 1), most congeners except for *Pylopaguropsis magnimanus* and *Pylopaguropsis pygmaeus*, have been documented to prefer hard bottoms in coral environments. Komai and Osawa (2004: 99) did note that some species usually “inhabit crevices of coral and rocky reefs or among large dead coral plates or blocks”; Osawa and Okuno (2007: 40) found that *Pylopaguropsis bellula* lives in “submarine caves or crevices on fore reef slopes”; and Arima (2014) documented that nine Japanese species (including one undescribed) preferred subtidal reefs and typically dark crevices on coral or rock bottoms and rocky walls.

The behavior of *Pylopaguropsis mollymullerae* sp. n. is also intriguing. Is there an ecological association of this new species with the “broad banded moray” or other moray species? Could this new hermit crab species function as a “cleaner” or a “den commensal”? At least in one instance, an individual was observed crawling on the body of a moray eel (Fig. 6E) with which this new hermit crab species seems to share a den. It is tempting to speculate that this individual of *Pylopaguropsis mollymullerae* sp. n. was feeding on mucus or other materials present on the body surface of the moray eel, and thus, this could be interpreted as a “cleaning” activity or existence of some kind of symbiotic relationship between hermit crab and moray. The brightly colored pattern with red stripes and setose antennae typical of most crustacean “cleaners”, tends to support this interpretation. However, as pointed out by Bauer (2004), cleaning symbiosis is a controversial topic, in part because the term “cleaner” has been applied based largely on anecdotal evidence to most of the 43 species of decapods that have been categorized as “cleaners”, all of which are caridean or stenopodidean shrimp except for two species of brachyurans (Spotte 1998, Côté 2000, Becker and Grutter 2004, Wicksten 2009). The term “cleaner” in the literature has been used for a wide range of presumed symbiotic associations that are not always clear, although studies have focused much more on “cleaner” fishes (e.g., Losey 1987 and references therein). It would be improbable for a hermit crab that carries a heavy shell as housing, to easily hop on hosts such as fishes that are most often suspended on the water column while “cleaner” activity is to take place. However, moray eels are typically in contact with the bottom inside their dens, and thus it would be possible for a hermit crab to access or climb on the body of the moray eel more easily, as observed for *Pylopaguropsis mollymullerae* sp. n. (Fig. 6E). Alternatively, the apparent preference of *Pylopaguropsis mollymullerae* sp. n. to inhabit caves alongside a moray eel, might indicate a case of “den commensalism” (as defined by Wicksten 2009) where the hermit crab can scavenge remains of food eaten by the moray eel. It would be of interest to conduct further, detailed studies on the behavior and ecology of *Pylopaguropsis mollymullerae* sp. n. to ascertain if any of the above interpretations are valid.

## Supplementary Material

XML Treatment for Pylopaguropsis mollymullerae

## Supplementary materials

Supplementary material 1 Photograph of live ovigerous female of *Pylopaguropsis mollymullerae* sp. n. stretching out from gastropod shell Data type: JPEG file Explanation note: Color photograph of *Pylopaguropsis mollymullerae* sp. n. taken by E Muller (dive 4125 095a) at dive site “Front Porch”: ovigerous female, not collected. This dataset is made available under the Open Database License (http://opendatacommons.org/licenses/odbl/1.0/). The Open Database License (ODbL) is a license agreement intended to allow users to freely share, modify, and use this Dataset while maintaining this same freedom for others, provided that the original source and author(s) are credited.Rafael Lemaitre

Supplementary material 2 Photograph of live specimen of *Pylopaguropsis mollymullerae* sp. n. in gastropod shell Data type: JPEG file Explanation note: Color photograph of *Pylopaguropsis mollymullerae* sp. n. taken by E Muller (dive 4125 156z2) at dive site “Front Porch”: sex not determined, not collected. This dataset is made available under the Open Database License (http://opendatacommons.org/licenses/odbl/1.0/). The Open Database License (ODbL) is a license agreement intended to allow users to freely share, modify, and use this Dataset while maintaining this same freedom for others, provided that the original source and author(s) are credited.Rafael Lemaitre

Supplementary material 3 Video of specimen of *Pylopaguropsis mollymullerae* sp. n. in gastropod shell, walking near crevice Data type: WMV Video file Explanation note: In situ video of *Pylopaguropsis mollymullerae* sp. n. taken by E Muller at dive site “Something Special”: sex not determined, not collected. This dataset is made available under the Open Database License (http://opendatacommons.org/licenses/odbl/1.0/). The Open Database License (ODbL) is a license agreement intended to allow users to freely share, modify, and use this Dataset while maintaining this same freedom for others, provided that the original source and author(s) are credited.Rafael Lemaitre
