# Supplementary figures and images for: Discovery of a new species of hermit crab of the genus Pylopaguropsis Alcock, 1905 from the Caribbean: “den commensal” or “cleaner”? (Crustacea, Anomura, Paguridae)
Source: Zookeys. 2017 Jan 19;(646):139–58. doi: 10.3897/zookeys.646.11132 (PMC5299445; doi:10.3897/zookeys.646.11132)

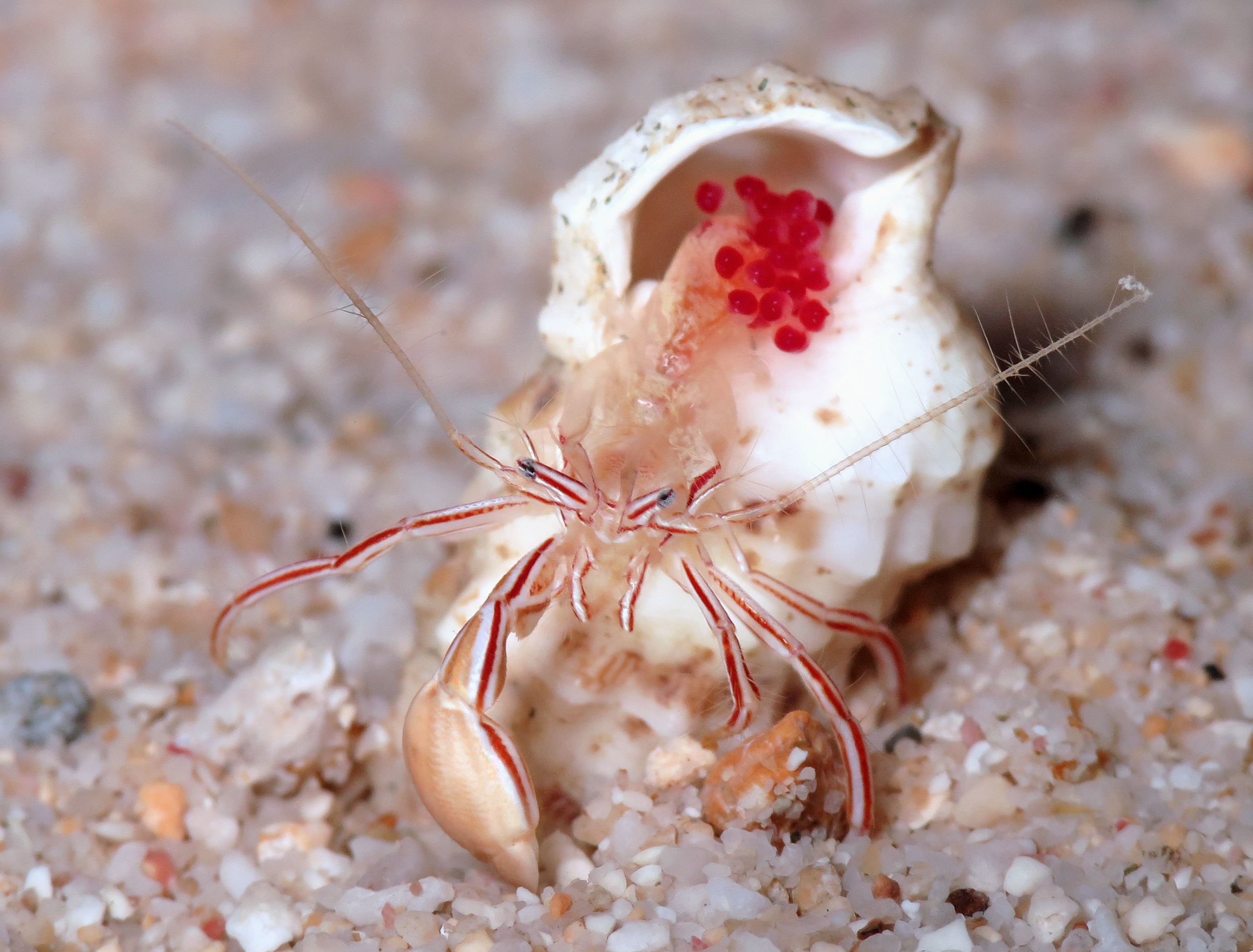

Supplement: Supplementary material 1 — Photograph of live ovigerous female of Pylopaguropsis mollymullerae sp. n. stretching out from gastropod shell [file zookeys-646-139-s001.jpg]

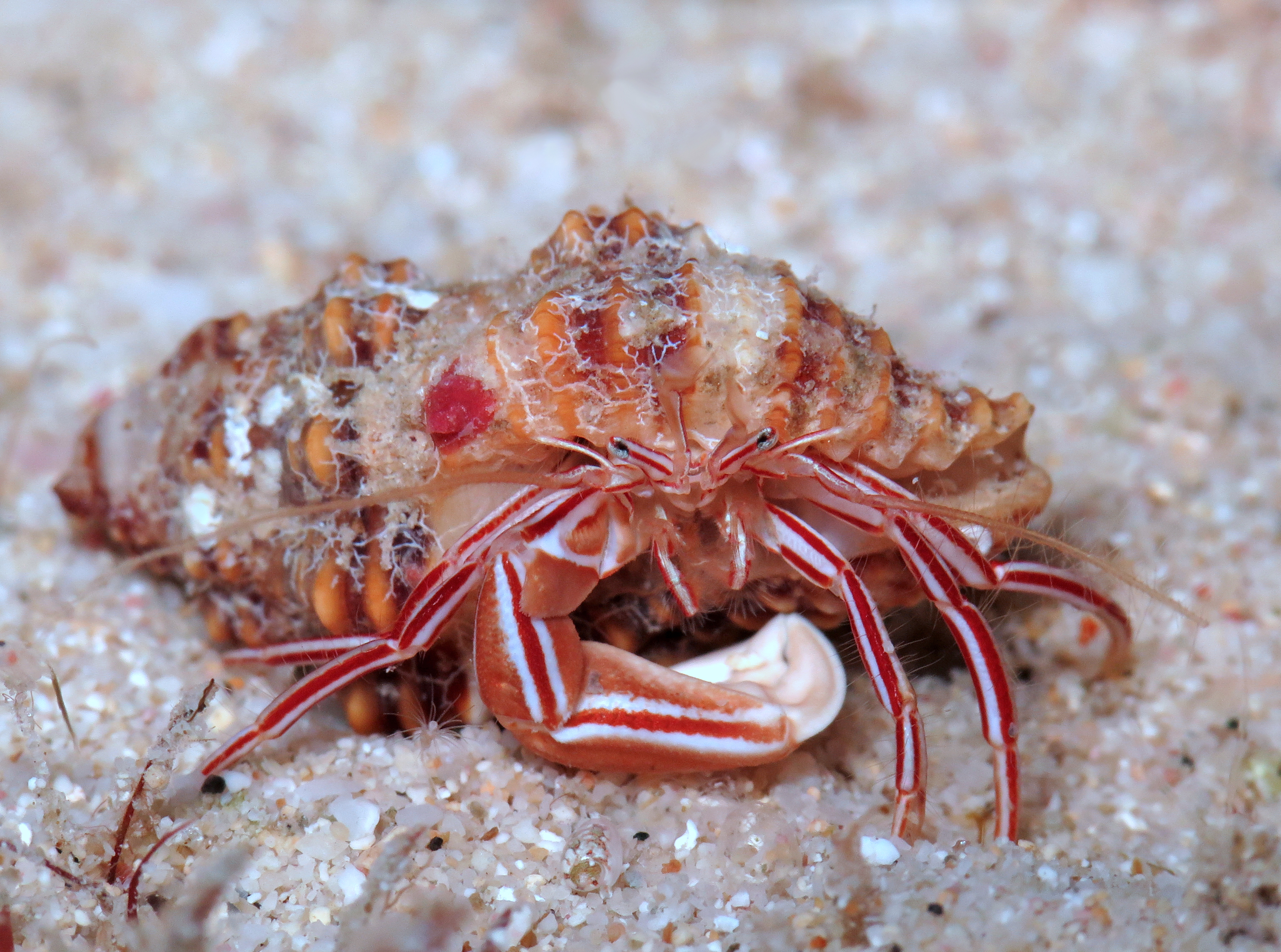

Supplement: Supplementary material 2 — Photograph of live specimen of Pylopaguropsis mollymullerae sp. n. in gastropod shell [file zookeys-646-139-s002.jpg]
